# Fusarium wilt of cotton may commonly result from the interaction of *Fusarium oxysporum* f. sp. *vasinfectum* with *Belonolaimus longicaudatus*

**DOI:** 10.21307/jofnem-2019-015

**Published:** 2019-04-23

**Authors:** Mychele B. da Silva, Richard F. Davis, Hung K. Doan, Robert L. Nichols, Robert C. Kemerait, Hannah C. Halpern, Marin T. Brewer, Ganpati Jagdale, Peng W. Chee

**Affiliations:** 1 Department of Plant Pathology, University of Georgia, Tifton, GA, 31793; 2 USDA–ARS Crop Protection and Management Research Unit, Tifton, GA, 31793; 3 Department of Plant Pathology, University of California – Davis, Davis, CA, 95616; 4 Cotton Incorporated, Cary, NC, 27513; 5 Department of Plant Pathology, University of Georgia, Athens, GA, 30602; 6 Department of Crop and Soil Sciences and Institute of Plant Breeding, Genetics, and Genomics, University of Georgia, Tifton, GA, 31793

**Keywords:** *Belonolaimus longicaudatus*, Cotton, Disease interaction, *Fusarium oxysporum* f. sp. *vasinfectum*, Fusarium wilt, *Gossypium hirsutum*, *Meloidogyne incognita*, Sting nematode, Survey

## Abstract

The interaction between *Fusarium oxysporum* f. sp. *vasinfectum* (Fov) and *Meloidogyne incognita* (root-knot nematode) resulting in Fusarium wilt (FW) of cotton is well-known. Although *Belonolaimus longicaudatus* (sting nematode) can also interact with Fov and cause FW, it has long been believed that virtually all of the FW in Georgia is caused by the interaction of Fov with *M. incognita*. In recent years, FW has been reported more frequently in Georgia, which suggests that something affecting the disease complex may have changed. In 2015 and 2016, a survey of 27 Georgia cotton fields in 10 counties was conducted. At least 10 soil and stem samples per field were collected from individual plants showing symptoms of FW to quantify plant-parasitic nematode levels and identify Fov races. Fov race 1 was identified in all samples in 2015, but one sample also had the LA110 genotype and another sample also had the LA108 genotype. In 2016, all Fov races and genotypes found in 2015 were present, however, MDS–12 and LA127/140 also were found. *Meloidogyne incognita* was present in 18% of fields in 2015 and 40% in 2016, whereas *B. longicaudatus* was present in all fields in 2015 and 75% of fields in 2016. Regardless of whether they occurred separately or together, *M. incognita* and *B. longicaudatus* were present, respectively, in 18% and 55% of individual samples in 2015 and 40% and 51% in 2016. However, *M. incognita* without *B. longicaudatus* was found in 7% of samples in 2015 and 34% in 2016, whereas *B. longicaudatus* without *M. incognita* was found in 45% of samples in 2015 and 44% in 2016. We conclude that Fov race 1 continues to be the dominant race in Georgia and many instances of FW in Georgia may be due to Fov interacting with *B. longicaudatus* and not *M. incognita* as previously believed.

*Fusarium oxysporum* is a widespread pathogen causing Fusarium wilt (FW) of numerous plant species, including watermelon, banana and cotton (Gordon and Martyn, 1997; Davis et al., 2006). Typical symptoms of FW are chlorosis of leaves and wilting of plants due to clogging of the xylem, which results in a characteristic vascular discoloration. *Fusarium oxysporum* is divided into formae speciales based on an isolate’s ability to reproduce on specific plant species. *Fusarium oxysporum* f. sp. *vasinfectum* (Fov) causes FW in cotton. FW incidence fluctuates greatly from year to year due to different management practices and environmental conditions (Davis et al., 2006; Hermanto et al., 2009; Lawrence et al., 2017). *Meloidogyne incognita*, the southern root-knot nematode, causes the greatest losses of any single pathogen of cotton in the USA (Lawrence et al., 2015), and the synergistic interaction of Fov with *M. incognita* that can result in greatly increased FW is well documented (Cooper and Brodie, 1963; Garber et al., 1979). Other plant-parasitic nematode species also have been reported to interact with *Fusarium oxysporum* to cause wilt, including *Belonolaimus longicaudatus* (sting nematode) and *Rotylenchulus reniformis* (reniform nematode) interacting with Fov in cotton and *Pratylenchus penetrans* (lesion nematode) interacting with *Fusarium oxysporum* f. sp. *pisi* on pea (Neal, 1954; Cooper and Brodie, 1963; Seinhorst and Kuniyasu, 1971). Although nematodes in several genera have been reported to be capable of increasing FW, the interaction of Fov with *M. incognita* is considered to be the most significant cause of FW in cotton (Rothrock et al., 2015) and the involvement of other nematodes is rarely considered.

Fov is divided into races, biotypes and genotypes. Genotypes are distinguished based on genetic variations among isolates. Isolates with morphological or physiological differences without described genetic differences are labeled biotypes (Downie, 2010). The race classification for Fov was originally based on pathogenicity of an isolate to different cotton cultivars or crop species. For example, race 1 caused disease on tobacco but not on soybean, whereas race 2 was capable of infecting both crops (Armstrong and Armstrong, 1958). Fov is genetically diverse and traditionally isolates were classified into eight nominal races, but they are now classified based only on DNA sequence, primarily partial sequence of *translation elongation factor*–*1α* (*EF*–*1α*) and the intergenic spacer (IGS) region of the rDNA (Cianchetta et al., 2015). The original race classification scheme that used host differentials to distinguish races is no longer used because the cultivars are not available and sequencing indicates that races 3 and 5 are the same, as are races 4 and 7, which also is supported by their being in the same vegetative compatibility group (VCG) (Skovgaard et al., 2001; Kim et al., 2005). Currently, six races of Fov that differ in pathogenicity and virulence to different cotton cultivars and other hosts are recognized worldwide. They include races 1, 2, 4/7, 3/5, 6, and 8. Other unique races have been identified in Australia and aggressive genotypes, including LA108, LA110, LA127/140 and MDS–12, have recently been identified in the southeastern USA. In Georgia, the most common races of Fov are 1, 2 and 8, and the most common genotypes are LA108 and LA110 (Holmes et al., 2009; Cianchetta et al., 2015). An “LA” isolate is a label originally used by Holmes et al. (2009) and such isolates have been shown to be in different VCGs within race 1 (Bell et al., 2017). Isolates within a race can have different levels of virulence (Glass et al., 2002).

In southern Georgia, awareness of FW among farmers, extension agents and consultants has increased greatly in recent years, as have yield losses attributed to this disease (Robert Kemerait, University of Georgia, personal observation). In fields infested with *M. incognita*, application of the widely used and effective nematicide aldicarb reduced both galling caused by the nematode and vascular discoloration and wilting caused by FW (Colyer et al., 1997). The phasing out of aldicarb (https://archive.epa.gov/pesticides/reregistration/web/html/aldicarb_fs.html; Kemerait et al., 2008) by its manufacturer (Bayer CropScience) from 2011 to 2016 may have allowed plant-parasitic nematodes to develop and reproduce in the fields more freely than when aldicarb was used. Although a formulation of aldicarb is now sold by another company in the USA, it is not currently widely used in cotton. The severity of FW is positively correlated with *M. incognita* population levels due to the interaction of the nematode and the fungus (Garber et al., 1979). Fov race 4 and Australian biotypes are unique because they can cause severe FW without interacting with nematodes (Kim et al., 2005), but those genotypes of Fov have not been found in the USA outside of California and Texas where Fov race 4 has been reported (Halpern et al., 2017). However, the movement of soil, water, equipment, plant material or seed could spread Fov race 4 or the Australian genotypes to other cotton-producing areas of the country. The reason for the recently increased incidence of FW in Georgia is not known. We conducted a survey of individual plants in cotton fields in southern Georgia showing symptoms of FW to determine which races of Fov were causing FW and which plant-parasitic nematode species were associated with the diseased plants.

## Material and Methods

Soil and plant samples were collected between June and November in 2015 and 2016 from cotton fields showing symptoms of FW. The survey included six counties and 11 fields in 2015 and seven counties and 17 fields in 2016 (Table 1). The Tifton fields 1, 4, and 5 in 2015 are the same as fields 3, 5, and 4 in 2016, respectively. County extension agents provided general information about soil proprieties such as soil texture, previous crop, and cotton varieties planted.

**Table 1:** County, cotton variety, soil description, planting date and sampling date for fields in Georgia sampled for *Fusarium oxysporum* f. sp. *vasinfectum* and nematodes in 2015 and 2016.

Year	County	Field	Cotton variety^a^	Soil description^b^	Planting date	Sampling date
2015						
	Ben Hill	1	DP 1252	Leefield loamy sand	May 15	August 13
	Berrien	1	DP 1252	Leefield loamy sand	April 24	August 28
	Cook	1	DP 1252	Stilson loamy sand	May 1	July 24
	Lowndes	1	DP 1050	Tifton loamy sand	May 8	August 28
	Tattnall	1	DP 1050	Loamy sandy	May 20	Mid-August
		2	DP 1050/DP 1137	Loamy sandy	May 15	Mid-August
	Tift	1	DP 1252/PHY 487/DP 1454	Stilson/Dothan loamy sand	May 4	June 24
		2	PHY 333	Ocilla loamy sand	June 2	August 28
		3	DP 1252	Ocilla loamy sand	unknown	July 10
		4	DP 1252/DP 1555	Dothan loamy sand	unknown	September 30
		5	DP 1454	Dothan loamy sand	May 7	June 19
2016						
	Coffee	1	ST 6182	Stilson loamy sandy	May 15	August 30
	Cook	1	DP 1252	Leefield/Irvington loamy sand	May 5	August 26
	Colquitt	1	DP 1538	Leefield loamy sand	April 20	August 31
		2	DP 1252	Dothan loamy sand	1st week of May	August 31
		3	DP 1553	Ocilla loamy fine sand	Mid May	August 31
		4	unknown	unknown	unknown	December 1
	Tattnall	1	DP 1558	Irvington loamy sand	May 13	September 22
		2	DP 1553/DP 1558	Pelham loamy sand	April 27	September 22
		3	DP 1553/DP 1558	Pelham loamy sand	April 29	September 22
	Tift	1	DP 1252	Ocilla loamy sand	April 24	August 24
		2	DP 1252	Dothan/Fuquay/ Tifton loamy sand	April 26	August 24
		3	DP 1252	Stilson/Dothan loamy sand	April 23	August 24
		4	ST 5115/ST 6182	Fuquay/Tifton loamy sand	June 1	August 26
		5	DP 1558	Dothan loamy sand	May 15	August 26
	Ware	1	PHY 444	Leefield loamy sand	May 18–20	September 7
		2	DP 1558	Pelham loamy sand	May 16	September 7
	Worth	1	DP 1558	Fuquay loamy sand	May 7	September 21

**Notes:** ^a^Cotton varieties names are abbreviated in the table; ^b^soil description information provided by county extension agents. Information is incomplete for some fields. Full names are as follows: DP 1252 B2RF, DP 1050 B2RF, DP 1137 B2RF, DP 1555 B2RF, DP 1454 NR B2RF, DP 1558 NR B2RF, DP 1538 B2XF, DP 1553 B2XF, PHY 487 WRF, PHY 333 WRF, PHY 444 WRF, ST 6182 GLT, and ST 5115 GLT.

In total, 8 to 15 samples were collected from each field. For each sample, a single plant showing symptoms of FW was arbitrarily selected, carefully uprooted, and the roots were observed for the presence or absence of root-knot nematode galls. The soil associated with the plant’s root system and a piece of the diseased plant’s stem from near the base were sealed in a plastic bag. The samples were stored at 10°C until processing. Nematodes were extracted from 150 cm^3^ of soil for each sample by the centrifugal flotation method (Jenkins, 1964), and the number of individuals for each genus of plant-parasitic nematode present was recorded.

For 2015 samples, Fov was isolated by disinfesting infected stem pieces in 0.875% NaOCl for 1 min, and then placing them into Komada selective medium (Komada, 1975) for five days. Five stem pieces per plant were cut open longitudinally and placed on a single plate. Individual colonies were then selected and transferred to a new plate containing Komada medium and allowed to grow for seven days. Then, single-spore isolations were made through successive serial dilution of conidial suspensions.

DNA was extracted separately from at least three different single-spore isolates per field. DNA extraction was accomplished by inoculating 125 ml flasks of potato dextrose broth with a mycelial plug (0.7 cm diameter) from the single-spore isolates and allowing them to grow for four days on a rotational shaker at 100 rpm. All the mycelium was then dried overnight on a sterile plate prior to using the modified DNA extraction method of Abd-Elsalam et al. (2003). Mycelium was macerated in liquid nitrogen and then put into 1.5 ml Eppendorf tubes prior to adding extraction buffer (200 mM Tris pH = 8.5, 250 mM NaCl, 25 mM EDTA, and 0.5% SDS). The sample was mixed on a vortex mixer and 4 µl of RNAase was added prior to incubation in a water bath for 65°C for 10 min. Then, 130 µl of 3 M sodium acetate (pH = 5.2) was added and the tubes were held at −20°C for 10 min. After centrifuging the sample for 15 min at 4,000 rpm, a 400 µl aliquot of the supernatant was collected and transferred to a 1.5 ml Epperndorf tube. The sample was then mixed in a 1:1 ratio with chloroform for 3 min and then centrifuged for 10 min at 8,000 rpm. The supernatant was removed and 650 µl of isopropanol was added to the tube to precipitate the DNA. Isopropanol was removed after centrifuging tubes for 10 min at 4,000 rpm. Finally, 300 µl of 70% ethanol was added into the tubes and centrifuged for 1 min at 4,000 rpm. Ethanol was removed from the tube and DNA allowed to dry overnight. Deionized water (100 µl) was added to rehydrate the DNA. The translation elongation factor (EF–1α) and IGS regions were PCR-amplified and sequenced for the Fov isolates as described by Kim et al. (2005) and Cianchetta et al. (2015). PCR reactions consisted of 12.5 μl of 2X Mean Green Master Mix (Syzygy Biotech; Grand Rapids, MI), 1 μl of each 10 μM primer and 2 μl of genomic DNA in a 25 μl reaction. Amplification reactions were performed in a thermocycler (PTC–100; MJ Research, Watertown, MA) under conditions as described by Cianchetta et al. (2015). PCR products were purified with a GeneJET PCR Purification Kit (Thermo Fisher Scientific, Inc., Waltham, MA) following the manufacturer’s instructions. A 24 μl sample of 40–68 ng/µl of PCR product and 12 μl of each 3 μM primer were sent to Quintara Biosciences (2600 Hilltop Dr, Building B, R332, Richmond, CA) for sequencing. Sequences were manually checked and aligned with known Fov sequences from GenBank database and private database to identify race or genotype.

For 2016 samples, Fov was isolated by washing cotton stems in soapy water, then surface sanitizing for 1 min in 95% ethanol, 2 min in sodium hypochlorite solution and 1 min in sterile deionized water. The outer layer of bark was removed, and small slivers of stem tissue were plated on acidified quarter-strength potato dextrose agar. Petri plates were incubated at 23°C with a 12-hr photoperiod for six to seven days. Single spores of cultures morphologically resembling *Fusarium* were transferred to quarter-strength potato dextrose agar under a dissecting microscope.

Genomic DNA was extracted from four single-spore isolates per field. Isolates were grown on potato dextrose agar overlain with sterile cellophane for six to seven days, after which time mycelia were harvested and lyophilized. Approximately 50 milligrams of lyophilized mycelia were placed in 2-ml microcentrifuge tubes with sterile glass beads, and macerated into a fine powder in a Geno/Grinder® (SPEX SamplePrep, Metuchen, NJ). DNA was extracted using a DNeasy Plant Mini kit (QIAGEN, Valencia, CA) following manufacturer protocol with the following modification: samples were eluted in 25 μl AE buffer (as opposed to 50 μl as stated in protocol) to increase the final concentration of DNA. PCR amplification, sequencing and genotyping were conducted as previously described.

## Results

**Fig. 1:**
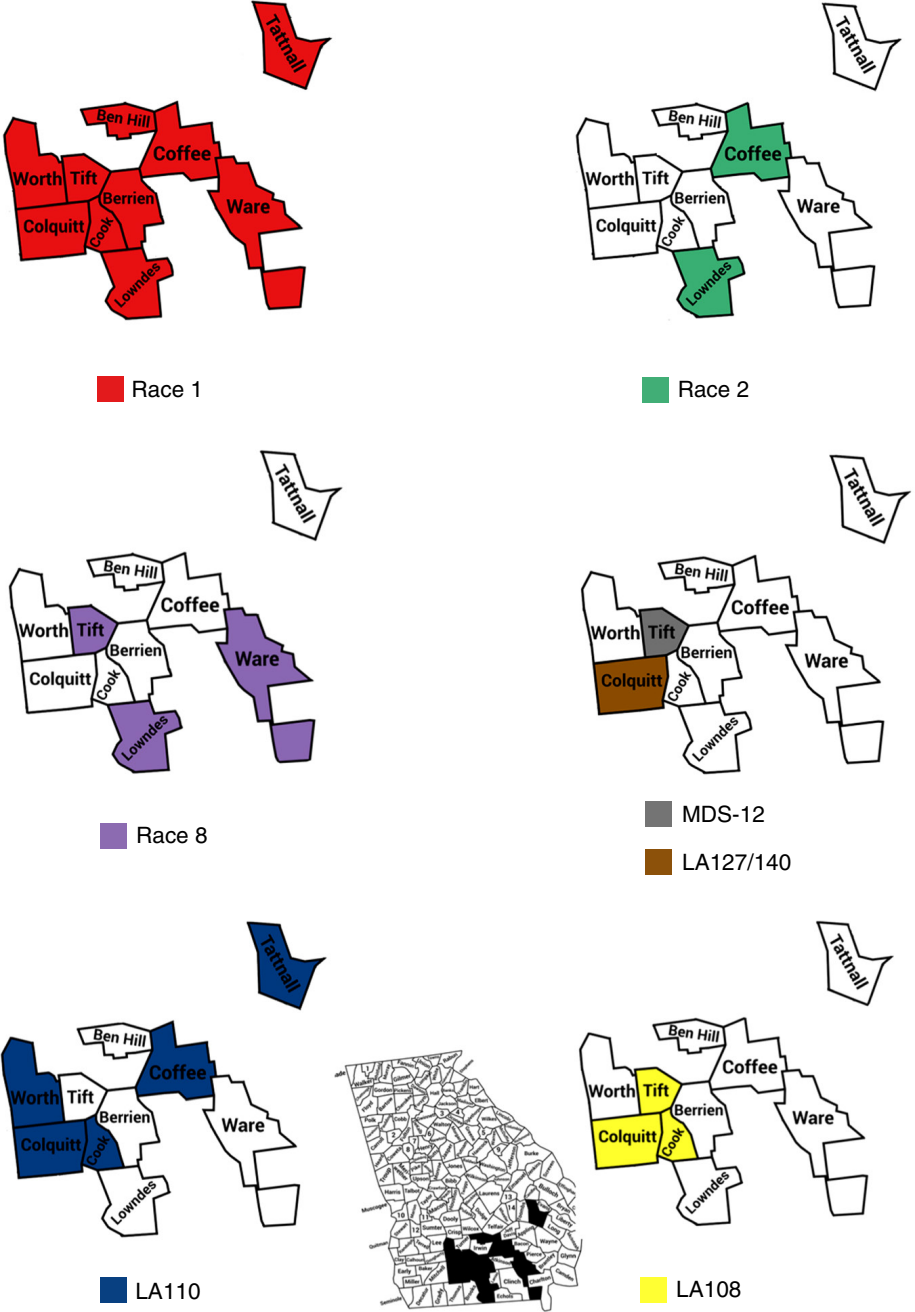
Map showing the location of the counties in Georgia that were sampled, and identification of which Fov races and genotypes were found in each county. Races and genotypes are distinguished by color.

Fov race 1 was found in all sampled counties in 2015 and 2016. In 2015, no previously unreported races or genotypes were found in Georgia. In addition to race 1, genotype LA108 was found in Lowndes County and LA110 was found in Tift County. In 2016, two new genotypes, LA127/140 and MDS–12, were identified in the fields sampled. Fov races 2 and 8 were found in Lowndes County; race 8 and genotypes LA127/140, LA110, and LA108 were found in Tift County; genotypes LA108 and LA110 were found in Cook County; genotypes LA108, LA110 and MDS–12 were found in Colquitt County; race 2 and genotype LA110 were found in Coffee County; genotype LA108 was found in Ware County; and genotype LA110 was found in Worth and Tattnall Counties (Table 2 and Figure 1).

**Table 2:** Fov races and plant-parasitic nematodes found in South Georgia fields in 2015 and 2016.

	Fov								Nematode species^a^							
County	Race/genotype description		*Meloidogyne incognita*		*Belonolaimus longicaudatus*		*Criconemella* spp.		*Pratylenchus brachyurus*		*Helicotylenchus* spp.		*Hoplolaimus columbus*		*Rotylenchulus reniformis*	
	2015	2016	2015	2016	2015	2016	2015	2016	2015	2016	2015	2016	2015	2016	2015	2016
Ben Hill	1	ns^2^	Y^3^	ns	Y	ns	Y	ns	Y	ns	N	ns	N	ns	N	ns
Berrien	1	ns	Y	ns	Y	ns	Y	ns	Y	ns	Y	ns	N	ns	N	ns
Coffee	ns	1, 2, LA110	ns	Y	ns	N	ns	Y	ns	Y	ns	N	ns	N	ns	N
Cook	1, LA108, LA110	1, LA110	N	N	Y	Y	N	Y	Y	Y	N	Y	N	N	N	N
Colquitt	ns	1, LA110, MDS–12, LA108	ns	Y	ns	Y	ns	Y	ns	Y	ns	Y	ns	N	ns	Y
Lowndes	1, 2, 8, LA108, LA110	ns	N	ns	Y	ns	Y	ns	Y	ns	Y	ns	N	ns	N	ns
Tattnall	1	1, LA110	N	N	Y	Y	Y	Y	N	Y	Y	Y	Y	Y	N	N
Tift	1,8, LA110, LA108	1,8, LA108, LA110, LA127/140	Y	Y	Y	Y	Y	Y	Y	Y	Y	N	N	N	N	N
Ware	ns	1, 8	ns	Y	ns	N	ns	Y	ns	Y	ns	N	ns	N	ns	N
Worth	ns	1, LA110	ns	Y	ns	Y	ns	Y	ns	Y	ns	N	ns	N	ns	N

**Notes:** ^a^ *Melodoigyne incognita*, *Belonolaimus* spp., *Pratylenchus brachyurus*, *Hoplolaimus columbus*, and *Rotylenchus* spp. have been reported to interact with *Fusarium oxysporum* f. sp. *vasinfectum* to increase Fusarium wilt. ns = not sampled; Y = yes (present); N = no (not present).

For the fields included in our survey in 2015, the predominant cotton cultivar planted was DP 1252 B2RF, which is susceptible to *M. incognita*. The *M. incognita*-resistant varieties planted in 2015 were DP 1454 NR B2RF and PHY 487 WRF, which were planted in 18% of the fields sampled. In 2016, the resistant variety DP 1558 NR B2RF was planted in 35% of the fields sampled (Table 1). *Belonolaimus longicaudatus* was present in all counties sampled in 2015 and in 70% of the counties in 2016 (Table 2). *Meloidogyne incognita* was found in 50% of the counties in 2015 and 70% in 2016. Other plant-parasitic nematodes associated with FW-damaged plants in our survey were *Pratylenchus brachyurus* (lesion), *Helicotylenchus* spp. (spiral), *Hoplolaimus columbus* (lance), *Criconemella* spp. (ring) and *Rotylenchulus reniformis* (reniform) (Table 2). Except for *R. reniformis*, the other genera were typically found at low population densities (data not shown).

The number of samples for each county in our survey (both years combined) is listed in Table 3. Only samples with one or more of the three species of nematodes reported (*M. incognita* and *B. longicaudatus*) or with likely potential (*P. brachyurus*) to interact with Fov to cause FW are listed. *Rotylenchulus reniformis* is not included because it was not found in 2015 and was found only in one field in 2016. A majority of the total survey samples (all counties for both years) contained *B. longicaudatus* either alone (34% of samples) or together with *P. brachyurus* (20%). *Pratylenchus brachyurus* and *M. incognita* were present in 7.3 and 6.6% of the samples, respectively, and both were present in 2.4% of samples. *Meloidogyne incognita* and *B. longicaudatus* were together in 2.4% of samples, all of which were in Tift and Worth counties. A greater number of samples were found with *B. longicaudatus* alone in Tattnall County (64% of samples) and in Ware County (65% of samples) than in the other counties (Table 3).

**Table 3:** Total number of samples infected with Fov collected from Georgia counties and the respective occurrence of *M. incognita*, *B. longicaudatus*, and *Pratylenchus brachyurus* in all combinations together or separately in the individual samples.

			Samples with concomitant occurrence of nematodes			
County	Total samples		*Meloidogyne incognita*	*Belonolaimus longicaudatus*	*Pratylenchus brachyurus*	All three
Ben Hill	10	*M. incognita*	1			0
		*B. longicaudatus*	0	6		
		*P. brachyurus*	0	1	0	
Berrien	10	*M. incognita*	0			0
		*B. longicaudatus*	0	6		
		*P. brachyurus*	0	1	4	
Coffee	8	*M. incognita*	4			0
		*B. longicaudatus*	0	0		
		*P. brachyurus*	4	0	0	
Cook	23	*M. incognita*	0			0
		*B. longicaudatus*	0	3		
		*P. brachyurus*	0	11	5	
Colquitt	40	*M. incognita*	9			0
		*B. longicaudatus*	0	1		
		*P. brachyurus*	16	8	2	
Lowndes	10	*M. incognita*	0			0
		*B. longicaudatus*	0	6		
		*P. brachyurus*	0	4	0	
Tattnall	50	*M. incognita*	0			0
		*B. longicaudatus*	0	32		
		*P. brachyurus*	0	6	0	
Tift	108	*M. incognita*	5			11
		*B. longicaudatus*	6	28		
		*P. brachyurus*	4	9	8	
Ware	20	*M. incognita*	0			0
		*B. longicaudatus*	0	13		
		*P. brachyurus*	0	7	0	
Worth	10	*M. incognita*	0			0
		*B. longicaudatus*	1	3		
		*P. brachyurus*	0	4	2	

For comparison to the results in our survey, samples from cotton fields in Georgia submitted to the Extension Nematology Laboratory at the University of Georgia from 2013 to 2016 were examined. During those four years, *B. longicaudatus* was detected in 1.2% of the samples from all counties throughout Georgia.

## Discussion

*Belonolaimus longicaudatus* can interact with Fov to cause FW in cotton (Cooper and Brodie, 1963), and control of *B. longicaudatus* can reduce FW in the field (Brodie and Hauser, 1970). Aldicarb was widely used for nematode control in cotton production before the production and sale of aldicarb by Bayer CropScience was phased out in the USA beginning in 2011 (https://archive.epa.gov/pesticides/reregistration/web/html/aldicarb_fs.html). Aldicarb is now produced and sold by another company (Ag Logic) in the USA, but it is no longer widely used. Although aldicarb was applied in cotton production primarily to manage *M. incognita* and *R. reniformis*, non-target nematode species including *B. longicaudatus* also would have been suppressed. Aldicarb is effective at reducing *B. longicaudatus* levels in the field (Rhoades, 1981; Weingartner and Shumaker, 1990). In our samples from plants with FW, *B. longicaudatus* was found much more frequently than expected based on the frequency that the nematode is found in samples from cotton fields submitted to the University of Georgia Extension Nematology Laboratory. The greatly decreased used of aldicarb and reliance on other nematode control tactics may have allowed *B. longicaudatus* to thrive, resulting in increased incidence and greater population levels of *B. longicaudatus*, and increases in B. longicaudatus levels could result in more interactions with Fov and greater FW incidence. Better control of *B. longicaudatus* should help reduce the incidence of FW. In fields where *B. longicaudatus* was not found, *M. incognita* was present. FW can be more severe in fields where both *B. longicaudatus* and *M. incognita* occur together (Yang et al., 1976). A few individual plant samples had neither nematode found associated with its roots, which could mean that neither nematode was involved in causing FW on that plant or that any nematodes involved were below our detection limit.

Race 1 was the predominant race of Fov in South Georgia in previous surveys (Holmes et al., 2009; Cianchetta et al., 2015). Additionally, races 2 and 8 and genotypes LA108 and LA110 also have been identified in Georgia (Holmes et al., 2009; Cianchetta et al., 2015). However, our survey is the first to report the presence of genotypes MDS–12 and LA127/140 in Georgia cotton fields. MDS–12 has been previously found in Alabama, which was the first report of this genotype in the USA (Bennett et al., 2013). Importantly, our survey did not find Fov race 4 or either of the two Australian biotypes, which cause severe FW without *M. incognita* infection (Kim et al., 2005).

Although the underlying mechanism of how *M. incognita* infection increases the severity of FW is unknown, it has been shown that *M. incognita* population levels positively correlate with FW incidence and severity (Garber et al., 1979). However, some fields in our survey in Berrien, Cook, Lowndes and Tattnall counties with high levels of FW did not have high levels of *M. incognita*, and the nematode was not detected in some fields. Most of our fields were infested with *B. longicaudatus*, suggesting that the Fov isolates were likely to be interacting with it to cause FW. Although other nematode species may form a disease complex with Fov (Cooper and Brodie, 1963, Seinhorst and Kuniyasu, 1971), our survey showed strong evidence of *B. longicaudatus* being the one most commonly associated with FW in Georgia cotton fields. Therefore, although a number of cotton cultivars with high levels of resistance to *M. incognita* are now available to growers, planting these cultivars may not provide adequate protection against FW in Georgia.

Another possible explanation for the recent increase in the incidence of FW in Georgia could be a genetic change in Fov allowing it to become more virulent. Fov race 4 is highly virulent on many cotton genotypes (Doan and Davis, 2014), and phylogenic trees have shown that LA110 and LA108 are genetically extremely similar to race 4, which may suggest that they share the high virulence of race 4 (Holmes et al., 2009). However, race 4 is reported to be a root-rot pathotype that caused disease following root-dip assays but not following stem injection and is therefore not considered a vascular-competent pathogen, whereas LA110 and LA108 are reported to be vascular-competent pathotypes that interact with nematode infection to increase disease severity in the field (Bell et al., 2017). The genetic differences between race 4 and genotypes LA108 and LA110 could be associated with differences in virulence or the need to interact with nematodes to cause severe disease under field conditions.

The interaction of Fov with nematodes other than *M. incognita* is not well documented, has been the subject of only limited research, and is seldom mentioned as a possible factor in FW incidence. The relatively common association of Fov with *B. longicaudatus* resulting in FW identified in our study requires additional research to be more fully understood. Better recognition that *B. longicaudatus* can interact with Fov to cause FW in cotton may lead to better control of FW by allowing growers to identify at-risk fields and minimize FW through better control of the nematode.
